# The effect of a portion size intervention on French fries consumption, plate waste, satiety and compensatory caloric intake: an on-campus restaurant experiment

**DOI:** 10.1186/s12937-018-0352-z

**Published:** 2018-04-13

**Authors:** Marie Vermote, Vickà Versele, Marijn Stok, Patrick Mullie, Eva D’Hondt, Benedicte Deforche, Peter Clarys, Tom Deliens

**Affiliations:** 1 Department of Movement and Sport Sciences, Vrije Universiteit Brussel, Pleinlaan 2, 1050 Brussels, Belgium; 2 Department of Interdisciplinary Social Sciences, Utrecht University, Heidelberglaan 1, 3584 CS Utrecht, Netherlands; 3 Department of Psychological Assessment and Health Psychology, University of Konstanz, Universitätsstraße 10, D-78464 Konstanz, Germany; 4 International Prevention Research Institute (iPRI), 15 chemin du Saquin, 69130 Ecully (Lyon), France; 5 Department of Public Health, Ghent University, De Pintelaan 185, 9000 Ghent, Belgium

**Keywords:** Portion size, Consumption, Plate waste, Satiety, Caloric intake, University, Nudging, Choice architecture

## Abstract

**Background:**

One of the driving factors of dietary overconsumption throughout the last decennia is the increase of food portion sizes. Larger portions induce higher daily energy intake, so reducing portion size may reduce intake of excess calories. However, real-life studies about the effects of portion size reduction are lacking. Therefore, this study examined the effect of a French fries portion size reduction on French fries consumption, French fries plate waste, satiety and caloric intake during the subsequent afternoon among university students and employees in a Belgian on-campus restaurant setting. Moreover, this study evaluated consumers’ perception about the portion size reduction.

**Methods:**

The study took place over a two-time (i.e. baseline and intervention week) 4-day period (Tuesday–Friday) in the on-campus restaurant where ±1200 meals are served every day. French fries’ portions were reduced by 20% by replacing the usual porcelain bowl served during the baseline week (±200 g) with smaller volume paper bags during the intervention week (±159 g) in a pre-post real-life experiment. French fries consumption and plate waste were measured in 2056 consumers at baseline and 2175 consumers at intervention. Additionally, interviews were conducted directly after lunch and again between 4 and 6 p.m. on the same day to assess satiety and caloric intake at pre and post in a small subsample of both French fries consumers (*n* = 19) and non-French fries consumers (*n* = 14). Post-intervention, the same subsample was interviewed about their perception of the portion size reduction (*n* = 28).

**Results:**

Total French fries intake decreased by 9.1%, and total plate waste decreased by 66.4%. No differences were found in satiety or caloric intake between baseline and intervention week among the French fries’ consumers. The majority (*n* = 24, 86%) of French fries consumers noticed the reduction in portion size during the intervention. Although most participants (*n* = 19, 68%) perceived the reduced portion size as sufficient, only a minority of participants (*n* = 9, 32%) indicated post-intervention that they would agree with a permanent implementation.

**Conclusions:**

Reducing portion size may lead to reduced caloric intake, without changing perceived levels of satiety.

## Background

Colleges and universities have the ability, like most educational institutions [1–5], to set up public health initiatives for their students via education, physical activity, but also via food environments [6–8]. In this perspective, they provide the ideal setting to promote healthy eating [6–8]. It has been demonstrated that, on average, college and university students gain a substantial amount of weight during their years at college or university [9, 10]. Research also shows that this weight gain coheres with poor diets, including low intakes of fruits and vegetables, insufficient variety in food consumption, and consumption of large portions [11, 12]. Moreover, it was found that students were more likely to gain weight when eating more frequently at the university restaurant [13]. A similar relation was found for those students frequently eating French fries [13]. Obviously, colleges and universities provide also services and employment for a large number of employees. Since the low intakes of fruits and vegetables and high fat intake is not only manifested in students, but also in the adult population in general [14], strategies that produce opportunities for healthy choices are also needed for employees [14]. Because adults spend most of their waking hours at work, the workplace can be a good setting for health related interventions [15]. When installing an intervention in a worksite cafeteria or restaurant a large number of people with varying age and socioeconomic status can be reached [16]. From this perspective dietary interventions conducted in a workplace setting are plentiful but not recent and little innovative, as most of workplace interventions used a labelling system or provided product information [14, 17–22]. However, few dietary interventions approached both students and employees simultaneously.

With regard to intervention development, manipulating the physical environment, may be recommended because one can easily and instantly reach larger groups of people [15]. According to Roy et al. [7] and Thorndike et al. [23], simple environmental interventions in food settings can lead to healthier food choices among (young) adults. Recently, environmental interventions in food settings are becoming very popular in the form of nudge and choice architecture studies [5, 24–27]. However, in order to have a chance of success, behavioural approaches must generally be ‘easy’ by reducing the effort to participate to a minimum [28].

A relatively easy environmental intervention, which has the potential to control people’s energy intake is portion size modification [29, 30]. Rolls et al. [31], for example, showed that portion size is positively related to daily energy intake. Beside the fact that (in general) people are consuming more kilocalories than they are expending [30], today’s portions of high-caloric foods contain larger quantities than recommended by dietary guidelines, even in Europe [32]. These findings raise the argument to decrease portion sizes in the battle against overweight and obesity.

Rolls et al. [31] proved that a reduction in portion size of 25% of all foods on two consecutive days led to a reduced food intake of approximately 10%. In their study, however, participants (i.e. randomly recruited young women) were invited in a laboratory setting, where the portion sizes and energy density of the foods of daily menus were changed. From a scalability and public health point of view, it is clear that confirmation by real-life setting trials is needed. To our knowledge, only one US study reduced the portion size of a calorie-dense food product (i.e. French fries) served in an on-campus university restaurant, presented in plain paper bags [33]. This experiment showed that reducing the portion size of French fries by 50% resulted in a 30% decrease in French fries consumption per consumer as well as 31% reduction in plate waste per consumer [33]. In an intercept survey, 70% of the 322 questioned French fries consumers indicated they did not notice the change in portion size [33]. In the latter study, consumers consisted primarily of US freshmen while dietary intake, and thus also dietary compensational effects during the rest of the day were not assessed. So, from this study we cannot conclude that portion size reduction will lead to reduced food intake, as the 50% reduction in French fries consumption may have been compensated for later on the day. In the laboratory experiment by Rolls et al. [34], ratings of hunger decreased as the size of the package containing potato chips increased, but this did not lead to a reduced energy intake at the subsequent dinner later that day. Such possible adjustments were also checked in a study by Jeffery et al. [35], where unannounced 24-h dietary recalls were conducted by phone after a 50% reduction of pre-packaged lunches in a naturalistic setting. Results showed that mean 24-h energy intake decreased with 278 kcal/day when a small lunch was served in comparison with a large one [35]. A British study investigating the effects of reducing breakfast both on hunger feelings and subsequent energy intake, showed no influence on subsequent energy intake but did show a significance difference in hunger feeling after reducing the portion with 40%. However, this study was conducted in an overweight population in a laboratory setting [36].

In summary, no real-life experimental studies including portion size modification assessed level of satiety and dietary compensational behaviour during the hours immediately after consumption. Since European research about portion size reduction is limited and US results cannot be extrapolated due to the different eating habits between both continents [37–39], more European studies are desirable. Therefore, the primary aim of the present study was to investigate the effect of a French fries portion size reduction on French fries consumption and plate waste among university students and employees in a Belgian on-campus university restaurant setting. Secondly, we aimed to investigate the effect of the intervention on level of satiety and caloric intake during the afternoon. Thirdly, we aimed to evaluate consumers’ perceptions about the portion size reduction.

## Methods

### Participants and design

The study was conducted in the on-campus restaurant of the Vrije Universiteit Brussel (Brussels, Belgium). The restaurant operates by a free-flow system which gives consumers the ability to choose daily between six different types of menus (i.e. menu 1 (generally meat dish), menu 2 (generally meat dish), fish-menu, vegetarian/vegan-menu, pasta menu, wok menu) or the salad bar. Consumers are free to choose a starchy side dish (French fries, mashed potatoes, boiled potatoes, or rice) when choosing one of the first four menus. Next to the main dish, a typical menu consists of soup of the day and a dessert (i.e. choice between fruit, yoghurt, pudding, cookies, or ice cream) and costs €5 for students and employees of the university, visitors pay a non-sponsored price (€10). French fries were chosen as the food of interest for the current study because it is high in calories, frequently chosen by students and employees, and relatively easy to manipulate in terms of portion sizes. Participants were university students and employees consuming one of the above mentioned menus as lunch at the on-campus restaurant during the experimental period. The on-campus restaurant is open on weekdays (Monday–Friday) from 11:30 a.m. till 1:45 p.m. and only serves lunch meals. Approximately 1200 to 1300 meals are served every day in the on-campus restaurant and all students and employees visiting the free-flow system of the on-campus restaurant were exposed to the experiment.

**Fig. 1:**
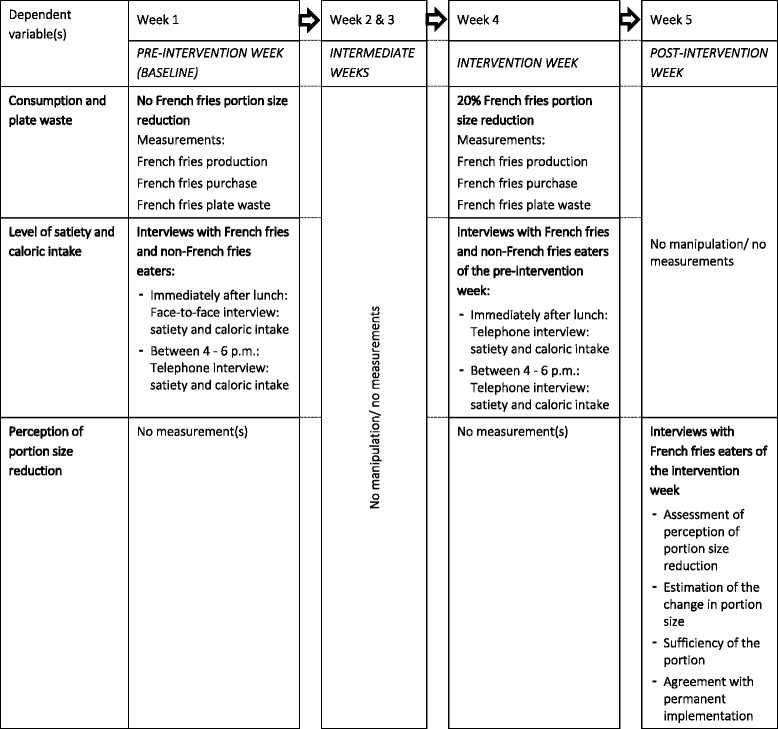
Flow diagram of the portion size experiment

The study consisted of a real-life experiment during which French fries consumption and French fries plate waste were measured in 2056 consumers during baseline week and 2175 consumers during intervention week. In addition, a convenient subsample of as much students and employees as possible (*n* = 296) was recruited on the spot to voluntarily participate in face-to-face and telephone interviews assessing level of satiety and dietary intake during lunch and later in the afternoon. Dietary intake was measured by means of a 4-h dietary recall. Dietary recall has been validated against a 4-day food record and provides a good overall ranking of intake [40]. These assessments were performed during both the pre-intervention and intervention week. Post intervention, students and employees who consumed French fries during the intervention week were asked about their perception of the portion size reduction in a post-intervention week. Figure 1 represents how the experiment was conducted. This study was approved by the medical ethics committee of the university hospital (Vrije Universiteit Brussel, Brussels, Belgium, B.U.N. 143201732012).

### Procedure

The experimental study took place over a two times (i.e. baseline and intervention week) 4-day period (Tuesday–Friday) during the opening hours of the on-campus restaurant (11:30 a.m. to 1:45 p.m.).

The course of the intervention was visually presented by Fig. 1. During the first (i.e. baseline) week, baseline data were collected of French fries consumption by registering the amount of portions of French fries (served in the usual porcelain bowls) purchased, the amount of French fries produced, and by weighing the wasted amount of French fries. Further, level of satiety shortly after completing lunch and caloric intake of the lunch were assessed using face-to-face interviews in a conveniently chosen group of students and employees immediately after having completed their lunch meal. Four hours after finishing these face-to-face interviews (between 4 and 6 p.m.), level of satiety of that exact moment and caloric intake and physical activity (as control for compensational behaviour for the larger/smaller portion) during the afternoon were assessed during telephone interviews within the same subgroup of students and employees who participated earlier that noon.

The second and third week were used as intermediate weeks during which no manipulations or assessments were carried out. This was due to the Easter holiday, during which the on-campus restaurant was less visited by students and employees.

**Fig. 2:**
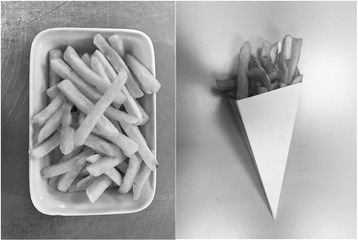
Comparison of the French fries served in the porcelain bowls and in the paper bags

During the fourth (i.e. intervention) week, French fries portion sizes were reduced by replacing the usual porcelain bowl containing approximately 200 g of French fries by smaller volume paper bags containing approximately 160 g French fries (Fig. 2), which corresponds to a reduction of 20%. Similar to the baseline week, French fries consumption, the amount of French fries produced and French fries plate waste were measured. Using telephone interviews, level of satiety and caloric intake were again assessed twice (right after lunch, and 4 h later) in the same subgroup of students and employees as in the baseline week. Because not everyone ate in the on-campus restaurant again, a part of this subgroup dropped out. At the end of the intervention week, two groups (French fries consumers and non-French fries consumers) were formed based on the side dish choice of the consumers. To avoid meal bias (i.e. some main dishes are more likely to go with French fries on the side than others), the same menus were provided during both baseline and intervention week.

In a fifth and last week (post intervention) all interviewed French fries consumers during the intervention week were asked about their perception of the portion size reduction.

### Measurements

#### Consumption and plate waste

Consumers needed to identify themselves at the cash registers by their student/employee card. Four to six observers registered whether or not French fries were chosen and if the menu was purchased by a student or employee. Two student assistants in the tray return area separated all un-eaten French fries from the total plate waste by throwing these into a separate bin. French fries waste was then determined by weighing the un-eaten French fries after restaurant closing-time.

#### Satiety and caloric intake

In the face-to-face interviews level of satiety was assessed shortly after completing their meal in the on-campus restaurant by means of the Satiety Labelled Intensity Magnitude (SLIM) scale. This scale consists of a vertical line scale including 11 labels describing different levels of hunger and fullness ranging from ‘greatest imaginable fullness’ to ‘greatest imaginable hunger’. Intermediate verbal labels were placed at the appropriate distance along the top and bottom of the scale in accordance with their transformed magnitude estimates [41]. In this way, every label gets a score from + 100 (greatest imaginable fullness) to − 100 (greatest imaginable hunger) [41]. During the interviews, consumers indicated which label of the SLIM scale best suited to their level of satiety. The labels were then converted to their respective scores as described by Cardello et al. [41].

Furthermore, consumers were asked about which meal and starchy side dish they had chosen, which dessert, whether or not they had chosen for soup and their choice of beverage. All food items consumed next to the lunch were reported as well. Leftovers were estimated in quantities in proportion to the normal portion served per container (e.g., bowls, cups, and glasses). Additionally, socioeconomic status (assessed for employees only), sex, age and cell phone number (to be able to re-contact the consumers later in the afternoon) were assessed.

Between 4 and 6 p.m. on the same day the interviewees were re-contacted by telephone and asked again to indicate their level of satiety by discussing the SLIM scale orally. Food and beverages intake during this interval were questioned (~ dietary recall), as control for compensational behaviour for the larger/smaller portion. This was done by questioning what and how much specifically they had eaten or drunk. Nubel (Nutrition analysis software program) was used to calculate the caloric value of the consumed food and beverages, by multiplying the estimated amount consumed (of a certain food/beverage product, in grams/ml) with the caloric values per gram/ml. Additionally, physical activity was questioned. This was done by asking the consumers to report the type and duration of any physical activity done between lunch and the moment of the phone call and whether or not they had sweated during this activity. Height and weight of interviewee were additionally questioned to calculate participants’ body mass index (BMI) and to be able to calculate the energy-expenditure of the physical activities by using ‘Appendix B: Energy expenditure in household, occupational, recreational, and sports activities’ of Katch, Katch, & McArdle William D [42].

Every consumer was phoned a maximum of three times on different times during this 2-h period before they were deemed to have dropped out. During the intervention week the same measurements were conducted in the same subgroup of students and employees, only if they again consumed a meal at the restaurant. A difference with the baseline week was that both interviews (i.e. directly after lunch and during the late afternoon) were conducted by telephone to be sure to include the same consumers as during baseline week. The time of ending the meal of the pre-intervention week was taken into account for the interviews directly after lunch in the intervention week. Again, everyone was phoned a maximum of three times on different times.

Only students and employees visiting the on-campus restaurant during both the baseline and intervention week were eligible to be allocated to the experimental or control group. The experimental group was formed by students and employees choosing French fries along with the same main dish on the same day of both weeks. In contrast, the control group consisted of students and employees choosing the same side dish different from French fries along with the same main dish on the same day of both weeks. Students and employees who chose a different main or side dish during the intervention week compared to the control week were excluded.

#### Perception of portion size reduction

During the fifth (i.e. post-intervention) week, all consumers of the experimental group were re-contacted by telephone. Consumers were asked whether or not they had noticed the reduced French fries portion size during the intervention week. If so, they were also asked to estimate the extent of alteration in portion size. Finally, participants were asked if the smaller portion was sufficient and whether or not a portion size reduction could be permanently implemented in the on-campus restaurant.

### Data analysis

During the preparation phase of the experiment the difference between fried and deep-frozen French fries was measured in order to be able to calculate how many kilograms of fried French fries came out of the deep-frozen French fries. The weight of fried French fries was equal to approximately 73% of the weight of deep-frozen French fries. French fries were fried for 3 min in vegetable oil (Vandemoortele® Risso Chef), consisting of palm oil, rapeseed oil, sunflower oil, and corn oil, at a temperature of 175 °C. Total French fries consumption (in kg) was calculated by subtracting the total plate waste (in kg) from the total amount produced (i.e. kg of deep-fried French fries used multiplied by 73%). Consumption per consumer was calculated by dividing the total French fries consumption by the number of French fries consumers. A similar calculation was done to determine portion waste. By dividing total French fries consumed per portion by the amount produced per portion, the ratio of consumed French fries per portion was calculated. A similar calculation was done to determine the ratio of waste per portion.

R version 3.4.0 and IBM SPSS Statistics 24 were used for data analyses. Since the proportions of French fries and plate waste were used as the outcome measures, a binomial test for one proportion (performed in R) was used to analyse differences in French fries consumption and plate waste between the baseline and intervention week. The same test was used to analyse whether or not the amount of consumers differed between the baseline and intervention week.

Drop-out analyses for the subgroup interviews were conducted using independent samples t-tests for baseline age and BMI and chi2 for sex. Repeated measures ANOVA mixed design was used to analyze differences in satiety and caloric intake between the baseline and intervention week in both the French fries consumers (i.e. experimental group) and the non-French fries consumers (i.e. control group). Furthermore, partial eta squared (partial η^2^) was calculated and reported as an estimate of effect size. Observed power (1-ß) was calculated as well. The statistical significance level was set at α = 0.05.

Descriptive statistics on portion size perception, satisfaction about the portion size, and feasibility of long-term implementation were calculated (in %). Finally, data (i.e. quotes) representing reasons why they would or would not agree with a permanent smaller portion were examined for recurrent instances and grouped together into similar categories.

## Results

A total of 4511 consumers were registered during the baseline week and 4868 during the intervention week. Of these consumers, 2056 (i.e. 45.6%) chose French fries during the baseline week, while 2175 (i.e. 44.7%) did during the intervention week. There was no significant difference in the relative number of consumers choosing French fries between the baseline or intervention week (*p* = 0.200).

### Consumption and plate waste

**Fig. 3:**
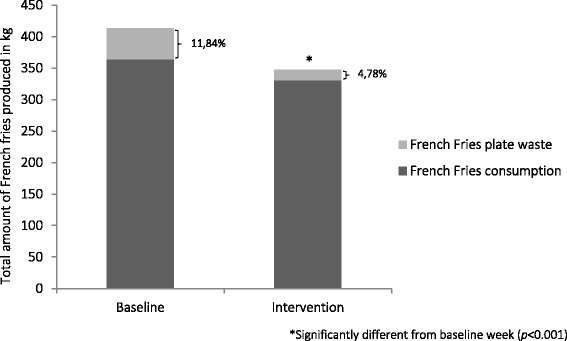
Changes in French fries consumption and plate waste between baseline and intervention week

Figure 3 shows the respective differences in French fries consumption and French fries plate waste between the baseline and intervention week. An overview of the effect of the portion size reduction on total French fries produced, consumed and wasted and the values for the individual portions can be found in Table 1. During the baseline week, consumers ate on average 88.1% (i.e. 177 g) of the available portion, whereas during the intervention week, consumers ate 95.2% (i.e. 152 g) of the available portion. Thus, although the absolute amount of French fries eaten was smaller in the intervention week, the proportion of the available portion eaten was greater.

**Table 1:** Effect of French fries portion size reduction on consumption and plate waste

	Average portion size (g)	Total consumers of the on-campus restaurant	French fries consumers	Total French fries produced (kg)	Total French fries wasted (kg)	Total French fries consumed (kg)	French fries consumption per portion (g)	French fries plate waste per portion (g)
Baseline week	201.0	4511	2056	413.3	49.1	364.2	177.1	23.9
Intervention week	159.8	4868	2175	347.5	16.5	331.0	152.2	7.6
*Difference*	*20.9%*			16.0%*	*66.4%*	9.1%*	*14.1%*	*68.3%*

*Significant differences between baseline and intervention week (*p* < 0.001)

### Subgroup interviews

**Fig. 4:**
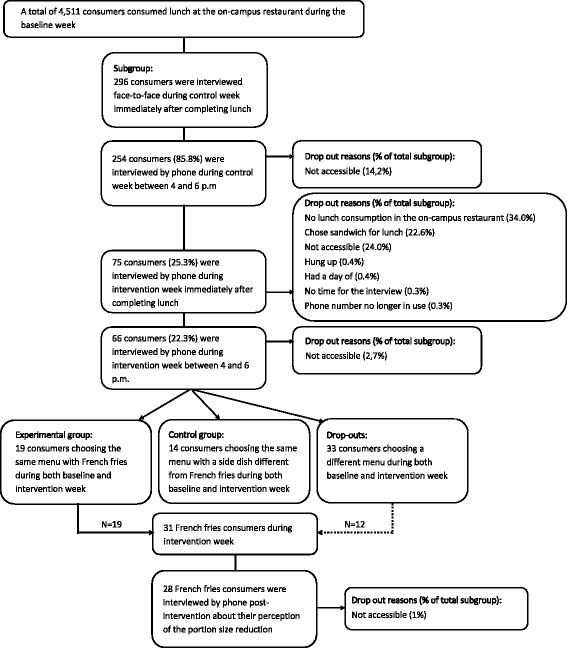
Flow chart of subgroup interviews

Table 2 gives an overview of the demographics and characteristics of the 296 consumers which were interviewed face-to-face during the baseline week immediately after completing their lunch at the on-campus restaurant. Figure 4 shows a flow chart of these subgroup interviews, together with drop-out reasons and shows that the final sample consisted of 66 participants (68.2% males).

**Table 2:** Demographics and experiment related characteristics of interviewees during baseline and intervention weeks (Mean ± SD,%)

	Baseline (*n* = 296)	Intervention (*n* = 66)
Sex (% males)	52.4	68.2
Age (years)	27.9 ± 12.1	30.9 ± 13.5
Occupation (% students)	60.8	43.9
BMI (kg/m^2^)	23.2 ± 3.6	23.6 ± 3.8
Underweight (%)	4.4	3.1
Normal weight (%)	72.3	69.2
Overweight or obese (%)	23.3	27.7
Food choice (%)		
French fries	48.3	50.0
Other	51.7	50.0

There were significant baseline differences in sex (chi^2^ = 8.519, *p* = 0.004) and age (*t* = 2.256, *p* = 0.025) between the retention (*n* = 66; 68.2% males; age = 30.9 ± 13.5 years) and the drop-out group (*n* = 230; 47.8% males; age = 27.1 ± 11.6 years), whereas no significant difference in BMI (*t* = 0.940, *p* = 0.348) was found between both groups (BMI retention = 23.6 ± 3.8 kg/m2, BMI drop-out = 23.1 ± 3.5 kg/m2).

Of the 66 people who remained to be interviewed in the late afternoon of the intervention week, only those who consumed the same menu with the same side dish during the baseline and intervention week were used for analysis (n_experimental group_ = 19; n_control group_ = 14). Consequently, 33 consumers were excluded for further analyses.

### Satiety and caloric intake

Table 3 shows the effect of the reduced French fries portion size on satiety and caloric intake during the afternoon. There was no significant interaction effect between the two groups (control and experimental) and the 2 weeks (baseline and intervention) for satiety (*F* = 0.034, *p* = 0.855, partial η^2^ = 0.001, 1-ß = 0.054) and caloric-intake (*F* = 3.176, *p* = 0.085, partial η^2^ = 0.093, 1-ß = 0.408). There were no significant differences between the experimental and control group concerning satiety (*F* = 1.578, *p* = 0.218, partial η^2^ = 0.048, 1-ß = 0.230) and caloric intake (*F* = 1.036, *p* = 0.317, partial η^2^ = 0.032, 1-ß = 0.167) and there were no significant differences between the baseline and intervention week in level of satiety (*F* = 0.025, *p* = 0.875, partial η^2^ = 0.001, 1-ß = 0.053) and caloric intake (*F* = 0.045, *p* = 0.834, partial η^2^ = 0.001, 1-ß = 0.055).

**Table 3:** Satiety and caloric intake during afternoon after portion size reduction (Mean ± SD, *F*-values, *p*-values)

	Baseline week	Intervention week	*F*_time x group_	*p*_time x group_	*F*_time_	*p*_time_
Satiety^a^			0.034	0.855		
Experimental group (*n* = 19)	23.1 ± 36.9	25.6 ± 36.8			0.071	0.793
Control group (*n* = 14)	10.9 ± 31.8	10.8 ± 40.7			0.000	0.987
Caloric intake^b^			3.176	0.085		
Experimental group (*n* = 19)	182.4 ± 319.0	84.5 ± 96.2			1.539	0.231
Control group (*n* = 14)	45.3 ± 69.2	122.4 ± 159.0			3.871	0.071

Experimental group *n* = 19; Control group *n* = 14. ^a^SLIM scale was divided as follows: greatest imaginable fullness = 100.0; extremely full = 79.4; very full = 74.3; moderately full = 46.7; slightly full = 31.9; neither hungry nor full = 0.0; slightly hungry = −18.6; moderately hungry = − 38.2; very hungry = −56.2; extremely hungry = −67.4; greatest imaginable hunger = −100.0. ^b^All the food and beverages consumed by the interviewee between their lunch and the time of the call between 4 and 6 p.m. in kcal

### Perception of portion size reduction

During the fifth and final week of the experiment, all French fries consumers interviewed during the intervention week (*n* = 31) were re-contacted. Because three participants could not be reached, 28 French fries consumers were interviewed about their perception of the portion size reduction. Of these 28 people, 86% noticed the smaller portion and, on average, estimated a reduction of 29.5 ± 10.6%. Respectively 14 and 54% of the participants reported that the reduced French fries portion size was more than enough or sufficient, while respectively 25 and 7% found the French fries portion size insufficient or largely insufficient. When the interviewer suggested a permanent implementation of smaller French fries portions, 18% completely disagreed, 29% disagreed, 21% were neutral, 25% agreed and 7% completely agreed. Commonly mentioned reasons for disagreeing were the absence of price adjustments (i.e. smaller portions should equalize lower prices) (*n* = 2), the reduced portion being too small (*n* = 7), the fact that people like to eat a lot of French fries (*n* = 1) and other reasons (*n* = 3). Commonly mentioned reasons for agreeing were that the smaller portion was enough (*n* = 4), the reduction of plate waste (*n* = 2), and the fact that a smaller portion is better for one’s health (*n* = 3).

## Discussion

This study aimed to investigate the effect of a French fries portion size reduction on French fries consumption and French fries plate waste among university students and employees in a Belgian on-campus restaurant setting. Secondly, this study aimed to investigate the effect of the intervention on satiety and caloric intake during the subsequent afternoon. Thirdly, we aimed to evaluate consumers’ perception about the portion size reduction.

Results showed that decreasing portion size was effective in reducing French fries consumption and French fries plate waste. A decrease of 20.9% in portion size resulted in a total French fries intake reduction of 9.1% and a relative decrease of 66.4% in French fries plate waste. Furthermore, level of satiety did not change when a smaller portion was served and consumers did not calorically compensate during the subsequent afternoon.

Our results are in line with other studies showing that portion size reduction can positively affect food intake. Although, compared to the study of Freedman et al. [33] in which a French fries portion size reduction by 50% resulted in a significant decrease by ±30% in French fries consumption and plate waste, our study showed that a smaller portion size reduction (i.e. 20.9%) results in a greater decrease in total plate waste (66.4%). However, it is important to mention that even a 9.1% reduction in French fries consumption can be useful to match the recommended dietary guidelines for total energy intake. On the other hand, reduction in French fries plate waste can be positive for the sustainability of the planet, since reducing food waste also reduces emissions of greenhouse gases [25]. Yet, it has to be mentioned that the use of paper bags in this intervention was not environmental friendly. When installing a permanent reduced portion, one should look for more sustainable alternatives.

The fact that the level of satiety of the French fries consumers in our study did not differ between the baseline and intervention week is in accordance with the study by Rolls et al. [31], in which consumers rated the portions as ‘smaller’ during the experimental week compared to the usual portion sizes, while ratings of fullness and hunger during both conditions did not differ. Also the absence of compensation at subsequent consumption after a portion size reduction is in line with previous research. Lewis et al. [36] found no differences in energy intake during lunch and the rest of the day after reducing the portion size of breakfast by 20 and 40% respectively. It should be mentioned, however, that the study of Lewis et al. [36] was conducted in an overweight population and in a laboratory setting. Further, Jeffery et al. [35] reported no compensation effects when consuming a lunch containing 1528 kcal compared to one of 767 kcal. It should be mentioned, however, that it is very important to interpret our findings with caution since the low statistical power observed. The results of the assessment of satiety and dietary intake can be false-negatives, since the great chance of type II errors. However, the observed effect sizes were very low too.

The results of the perception interviews, where 86% of the consumers noticed the smaller portion size, are not in line with those found by Freedman et al. [33], in which only 30% noticed the smaller portion. This difference could be due to the change of container between baseline and intervention week in our study, or due to the fact that the interviews made the consumers more aware of the portion size intervention.

A permanent implementation of a smaller portion may be recommended to health promoters, university policy makers and restaurant operators, since 68% of the French fries consumers in the present study had sufficient or more than enough with the smaller portions.

A strength of the present study is that we used a real-life setting which maximises the external validity of the intervention. Secondly, we anticipated that consumers would feel that certain main dishes matched better with French fries than with rice or (mashed) potatoes. Therefore, we excluded meal bias by providing the same menus during the baseline and intervention week. Furthermore, to counter testing bias, we did not communicate about the content and the duration of the portion size experiment. However, the majority of consumers did notice the smaller portion and the unusual paper bags, which could have influenced consumers’ food choice anyway. But our results showed that there was no difference in the amount of French fries portions sold per capita between the baseline and the intervention week. Finally, we checked for compensation effects during the subsequent afternoon. Although some studies already showed that there were no compensational effects seen after a portion size increase [35] or decrease [36], our study was, to our knowledge, the first real-life experimental study taking caloric intake throughout the subsequent afternoon into account when reducing portion size.

The first and possibly most important limitation of this study was the focus on consumption and plate waste of French fries and not on other food groups. Measuring plate waste of the whole lunch could have given us insights in the compensational behaviour of consumers during lunch, i.e. it may be that participants consumed more of the other food items of the chosen lunch when reducing French fries portion. Rolls et al. [31], for example, showed that focusing on several foods may be effective in monitoring consumption and energy intake. Unfortunately, it was practically impossible to measure plate waste of the whole lunch because of the difficulty of separating the wastes of every food product for French fries vs. non-French fries consumers. Secondly, although the present study found no compensational effects during the afternoon, participants may have compensated for the reduction in portion size by eating more during dinner. Therefore, it may be interesting to include this in future studies. However, several studies showed no compensational behaviour in subsequent meals after portion size modifications [31, 35, 36]. A third limitation of the present study is that we did not include a control setting. Because there are no similar on-campus restaurants available with comparable food availability, this was not possible. Also, for the measurement of French fries consumption and French fries plate waste in this study, it was not feasible to include a control group of students and employees because it was practically impossible to offer one part of the consumers less French fries (in the paper bags) while another part would still receive the usual portion (in the porcelain bowls). Lack of control may have jeopardized the internal validity of our study. Fourthly, our intervention was of short duration (i.e. only 1 week) and no follow-up measurements were conducted. Therefore, it is not clear whether this intervention effect would sustain in the long run. One study showed that the opposite, i.e. increasing the portion size of lunch with 50% over a longer period of time (4 weeks), resulted in a higher energy intake during the day, without compensation over time [35]. It should be noted, however, that 4 weeks can hardly be classified as “long-term”. It is clear that more studies investigating the long-term effectiveness of portion size reductions are needed. Fifthly, it must be mentioned that for the subgroup interviews drop-out was large, causing a small sample size for the comparisons of satiety and dietary (caloric) compensation effects. This may have caused selection bias. For example, it may be that only those with a particular interest in health remained in the study. In contrast to sex and age, however, there was no difference in BMI between the retention and drop-out group. The above limitation shows the difficulty and complexity of such real-life trials and should be taken into account when conducting future research. It can be said that future studies with larger sample sizes are needed to confirm our results. Furthermore, it must be repeated that the interpretation of the results of the assessment of satiety and dietary intake should be taken with caution, since the small subsample may be the reason for the low statistical power. Future research should take this in mind and should try to work with sufficiently large samples, to obtain more statistical power. Sixthly, concerning consumption and plate waste, it was not feasible to investigate differences between students and employees, because it was practically impossible to weigh every paper bag sold to students versus employees. Also, separating students’ plate waste from that of employees was not feasible. Seventh, the price of the reduced portion of French fries was not adjusted, which may have been of influence on the choice of the consumer to choose French fries or not. Especially, students, who are more sensitive to price changes than employees, could have opted a side dish different from French fries during the intervention week. However, since there was no significant difference in the number of consumers choosing French fries (and the proportion of students within these French fries consumers) between the baseline or intervention week, a lack of price adjustment was probably not crucial for the consumers’ choice. However, the lack of price adjustment came up for discussion in the perception interview, when asking why people would or would not agree with a permanent implementation. The share of consumers indicating this as stumbling block was minimal, as only two out of 28 interviewees addressed this reason to disagree with a permanent implementation. Attractiveness of the French fries served in paper bags could have influenced the consumers’ choice. Introducing paper bags before the baseline week could have ruled out a possible attractiveness effect ensuring a ‘true’ effect of the reduced portion was observed. However, no significant difference in the number of consumers choosing French fries between the baseline and intervention week was found. Misinterpretation of the terms satiety and fullness could also have influenced our results. Since satiety was questioned as a rating of fullness in the face-to-face interview, consumers could have reported a full feeling after consuming a 50 cl bottle of diet soda or water, which would not have corresponded with the calculated caloric intake. However, the study of Tremblay et al. [43] concludes that the use of VAS (visual analogue scale) scores, using ratings of hunger or fullness, has high relevance and is a simple and valid tool to predict variations in energy intake. Finally, this study only focused on one single energy dense food product and did not investigate possible effects on other foods. Also, focusing on different food products could have given us insight into which kind of portion size reduction consumers are most susceptible for. Therefore, future studies should examine whether portion size reduction of other foods will have similar effects and whether or not decreasing portion size of one food has an effect on the intake of other foods.

## Conclusion

Reducing the portion size of French fries was effective in reducing French fries intake and French fries plate waste in an on-campus restaurant setting. More specifically, a portion size reduction by 20.9% resulted in a reduction by 9.1% in total French fries intake and a reduction by 66.4% for total French fries plate waste. Satiety of the consumers did not change and no dietary compensation effects were observed. A large part of French fries consumers noticed the reduced portion size. Although the majority thought that the portion size was sufficient, only a minority agreed with a permanent implementation. Changing portion size is a relatively easy and promising environmental strategy which may lead to a more balanced/healthier food intake.

## Abbreviations

BMI: Body mass index
SLIM: Satiety Labelled Intensity Magnitude
